# On the Permanent Evidences of Successful Vaccination

**Published:** 1827-05

**Authors:** G. Gregory

**Affiliations:** Physician to the Small-Pox and Vaccination Hospital.


### SMALL-POX AND VACCINATION.

*On the Permanent Evidences of Successful Vaccination.* By G. Gregory, M.D. Physician to the Small-Pox and Vaccination Hospital.

In offering a few observations on the phenomena of vaccination, and more especially on the appearances of vaccine cicatrices, I am fully sensible that I enter on a field of investigation where very little remains to be gleaned. The scientific labours of Jenner and Willan, (not to mention a host of authors of lesser name,) have almost, if not altogether, exhausted the subject; and, under such circumstances, it may reasonably be asked, why I thus recur to it with such small prospect of novelty or benefit? My only excuse is the earnest hope that it may prove useful—first, to refresh the memory on certain minute points connected with vaccination; and, secondly, to show that the lapse of twenty years has only served to strengthen the conclusions of the best early writers.

No reasonable doubt, I presume, can be entertained that the occurrence of small-pox after vaccination is considerably more common than that of small-pox after variolous inoculation. Could it be made to appear that the one was not more frequent than the other, we should at once have a satisfactory explanation of the circumstance in the theory of idiosyncrasy, or "*peculiar susceptibility in the body of the variolous poison.*" That this principle is sufficient to account for a certain proportion of the cases of small-pox which occur after vaccination, appears to be clearly and indisputably established; and hence it is that the arms of some of those who thus suffer will be found, on examination, to exhibit the most perfect characteristics of a vaccine cicatrix. When, however, the disease *does* occur under such circumstances, it will, with very few exceptions, differ so materially in its symptoms and progress from that of common small-pox, that it may, with perfect propriety, and in strict accordance with the language of the old authors in physic, receive the name of Variolæ Spuriæ, Bastard Small-Pox, Chicken-Pox, or Varicella. In all its characters, it corresponds perfectly with the mild disorder (so denominated) under which the inoculated still occasionally suffer, as they used formerly to do before vaccination was known or thought of. The following case may be taken as an illustration.

Case I.—Lieutenant Wm. Gregory, of the Royal Engineers, (the author's brother,) was inoculated for small-pox at Canterbury, in the year 1794. He had the disease with rather more than usual severity, and for two or three days some little uneasiness was felt for him. Twenty-nine years afterwards,—viz. in January 1823,—after a long residence in Nova Scotia, he accidentally visited the Small-Pox Hospital in my company, and was at the bedside of several patients labouring under a very malignant form of small-pox. In about a week afterwards, he was unexpectedly attacked with rigors, headache, and other marks of fever. On the third day an eruption of papulæ took place, which ran the usual course of highly modified small-pox,—in other words, of chicken-pox. The pock turned upon the fourth or fifth day, and in less than ten days afterwards he was perfectly well, and following his usual avocations.

The disease resembled, in every respect, the milder forms of eruption which are now so common after vaccination. I feel perfectly convinced that similar cases were very frequent before the discovery of the cow-pox, but they excited less attention than now, on account of the greater comparative prevalence of true small-pox, natural and inoculated. To this form of chicken-pox I would venture to apply the term Varicella Varioloides, in contradistinction to the "*varicella symphatica*" of Willan.

While I thus express my perfect conviction of the sufficiency of the principle of idiosyncrasy to explain many cases of small-pox after vaccination, whether complete or incomplete, I must acknowledge, at the same time, my belief that the excess of such cases over those of secondary small-pox, is explicable only on the theory of *imperfect vaccination,*—that is to say, on the principle that the system may be partially and imperfectly saturated with the vaccine virus, giving thereby not a complete and permanent, but a partial and temporary security. This doctrine is that of Dr. Jenner, who acknowledges, however, that it was first given to the world by Mr. Dunning, of Plymouth.* Dr. Jenner, speaking of the varieties in the appearance of the vaccine vesicle, states that they exist in all degrees, "*from those trifling deviations which prove no impediment to the vaccine security, up to that point of imperfection in the pustule which affords no security at all.*"—"In saying *no security at all,*" adds Dr. Jenner, "I perhaps commit an error; for it strikes me that the constitution loses its susceptibility of small-pox contagion, and its capability of producing the disease in its perfect and ordinary state, in proportion to the degree of perfection which the vaccine vesicle has put on in its progress; and that the small-pox, if taken subsequently, is modified accordingly." These were Dr. Jenner's views on the 23d February, 1806; and that his further experience, corroborated then, is proved from this, that they are reiterated and forcibly urged in a circular letter which I received from him on the 28th May, 1821, within less than two years of his death.

The principle appears to me to be perfectly correct, and in fact to be the most important of all those which influence the production of small-pox in a large proportion of mankind at the present day. It may be thus more fully developed. Perfect vaccination affords, with certain exceptions, complete and permanent security against the small-pox. Imperfect vaccination affords security more or less permanent, more or less complete, according to the degree of imperfection in the vesicle, and the consequent saturation of the system. The whole extent of my experience at the Small-Pox Hospital for

* See Willan on Vaccine Inoculation, 4to. 1806; page v. of the Appendix.

the last five years, my observations on the phenomena of re-vaccination, and the results of my correspondence with practitioners in the country, bear out this principle in its fullest extent.

Occasional exceptions to this, as well as to any other great pathological doctrine, have doubtless occurred, and may therefore occur again, to warn us how we tie down nature too closely to rules; but the general principle still remains untouched.

Presuming it, then, to be well ascertained, the question comes to be, *what are the evidences of perfect vaccination ?* and how can we venture to pronounce an individual safe from the perils and dangers of small-pox? The evidences of vaccination are of two kinds,—the temporary and the permanent. The former (in other words, the appearances of the vesicle,) are so admirably detailed by Jenner and Willan in their respective works, that it would be quite unnecessary to repeat them here. The latter have attracted less attention, but are not the less deserving of a full investigation. I mean the various appearances of vaccine cicatrices. To point out these, and to show with what modifications they are to be received as evidences of the purity and perfection of the vaccine process, will be my object during the remainder of this paper.

1. A true and perfect vaccine scar should be distinctly defined, even after the lapse of twenty years,—that is to say, the specific inflammation should have penetrated the cutis vera to such a depth that the scar which results is clearly to be traced in all periods of after-life. In order that this should take place, it is nearly indispensable that the scab should remain on—or, at any rate, that cicatrisation of the sore should not be completed, prior to the twenty-first day. I have seen, however, cases where the cicatrix was formed as early as the fourteenth or fifteenth day. In all such cases the impression of the scar wears out in the course of time, the vaccination is imperfect, and the system is either partially or, after the lapse of a certain number of years, wholly open to the attacks of small-pox.

2. A true and perfect vaccine scar should be circular, or nearly so. That is to say, the specific inflammation should not have been superseded by common inflammation. Should this, indeed, take place *late* in the disease, the scar will be irregular, but the vaccination may still be perfect. When, however, common inflammation supervenes early, the scar is irregular in its form, the vaccination is incomplete, and the system remains susceptible of small-pox, more or less modified, according to the degree of perfection which the vaccine vesicle may previously have attained. The diameter of the scar, provided that scar be circular, is of little moment. That of a sixpence or small wafer appears to me to be the largest which is compatible with complete security.

3d. The perfect vaccine scar should be indented and radiated. It may be proper to premise that the vaccine vesicle has a regular organisation (consisting of cells tied down by a central band) like that of variola; and it is certainly a mark of the perfection of the vaccine process, when the indentations and striæ of the original cells remain to testify that the vesicle was uninjured in its progress. I am very ready to acknowledge, however, that these marks are not essential to the perfection of the vaccine cicatrix. I have seen several cases of the most highly modified chicken-pox after vaccination, where such characters in the scar have not been perceptible.

I proceed next to point out, in a summary way, the sources of such irregularities in vaccination as lead to imperfect cicatrices, and to partial or temporary saturation of the system.

1. The first is the employment of effete virus,—of virus, that is to say, taken at too late a period of the disease; of virus injured by heat, by long keeping, or by the rust of a lancet. The vaccine fluid, as Willan long ago remarked, is the most delicate of all the morbid poisons, and is the most easily altered in its qualities. I have never vaccinated with points and glasses since my attention was turned to the subject; and, however I may feel the necessity that (in the country sometimes) exists for this measure, I am strongly convinced of the benefit that would arise from vaccinating, in all cases whatever, with recent lymph, taken not later than the eighth day.

2. The second source of imperfection in the vaccine vesicle and subsequent cicatrix, is the pre-occupation of the system by some important process, such as teething, or by some actual disease, such as inflammation of an important viscus, fever, hooping-cough, porrigo favosa, or herpes. In such a state of body as this, the vesicle is apt to be converted into a pustule, and the specific to be superseded by common inflammation. The following case may be taken as an illustration.

Case II.—In the summer of 1823, I was requested to visit a girl in Spa-fields, labouring under severe small-pox subsequent to vaccination. She was one of a family of five, all of whom had been vaccinated at the Small-Pox Hospital. I found the patient with a load of small-pox, which were afterwards slightly modified in their progress. The patient, however, happily recovered. The scar upon the arm in this case was large and irregular; and the mother told me that the inflammation during the vaccination was so severe, that she was obliged to cut the infant's dress, and to keep the arm constantly wetted with cold lotion. Notwithstanding which, Mr. Wachsel, then resident surgeon of the hospital, gave it as his opinion that the process was staisfactory. The scars upon the arms of the other children were perfect, and they all remained in the same room with their sister during her illness, without suffering in the slightest degree.

3. The third, and the only other source of imperfect vaccination with which I am acquainted, is the institution of the process at too advanced a period of life. All those who are in the habit of vaccinating must know how much more complete the vesicle appears in healthy children between the second and fourth month after birth, than at later periods of life; and, in fact, how difficult it is to get perfect appearances in adults. The principle I believe to be this:—In very early life, the extent of inflammation upon the arm bears a much larger proportion to the rest of the body than at an advanced age; and it is reasonable to presume that the system will be more thoroughly imbued with the vaccine virus when the mass of blood and humours is small, than when it is great. This affords a second reason why the mode of town vaccination is so much superior to that of the country.

In London, and I presume in all large towns, children are regularly brought to be vaccinated as they attain the age of three or four months: whereas, in the country, periodical vaccinations of parishes take place, without reference to age.

I would wish to offer one observation with reference to the gradations in the modifying influence of imperfect vaccination. It is, I believe, contended by some (even of high authority in the profession,) that vaccination will always secure the constitution from the loss of life: in other words, that the degeneracy of vaccine saturation stops short at that point at which life is put in danger. My experience does not warrant me in subscribing to this position. I believe the cases to be rare, but yet in practice it will be found that there are imperfections in the process of vaccination, which, after the lapse of a certain time, leave the system as completely open to the full influence of the variolous poison as if vaccination had never been practised. In illustration of this remark, I refer to a paper which has appeared in a late Number of the London Medical and Physical Journal.

I have already stated, that my observations on *revaccination,* as well as the results of my correspondence with country practitioners, equally bear me out in these views. I select the following from my note-book as a specimen of the former; and, with respect to the latter, I shall prefix a brief notice from J. DALTON, Esq. an eminent surgeon of Bury St. Edmunds, of the epidemic small-pox which occurred in that town in 1825. The remarks of this gentleman, written without knowledge of, or reference to, my own ideas on the question, will, I think, be found peculiarly deserving of attention.

CASES III. AND IV.—On the 22d July, 1822, I revaccinated Miss M—, second daughter of Lord D—, and at the same time a maid-servant in the family of the Countess of Denbigh. The lymph which I employed was from a most perfect vaccine vesicle (seventh day), without the slightest surrounding areola, and from a beautiful and healthy child.

The young lady (being then fourteen years of age,) had been vaccinated with great care by Dr. Jenner. The scar upon her arm was circular, well defined, and in every respect perfect. The maid had been vaccinated by a country practitioner, without any particular precaution. The scar upon her arm was large, oblong, and irregular, with a raised line running down its centre, as if considerable and extensive ulceration had once existed there.

In the first case, the revaccination caused only a slight irregular inflammation, subsiding entirely on the fourth day. In the latter case, the vaccination advanced, though in a modified way. An extensive areola formed on the sixth day. On the eighth, the areola was subsiding, but the arm was still considerably inflamed. The vesicles had began to desiccate. There was also present considerable swelling of the axillary glands, with pain.

Is it not probable, from the result of these cases, that the maid-servant would have taken small-pox had she been exposed to it, and that the young lady would not?—that in the one case the constitution was saturated with cow-pox, and in the other that it was not?

I shall conclude with Mr. Dalton's communication.

*On Small-Pox as it prevailed epidemically at Bury St. Edmunds, in* 1825. By J. DALTON, Esq. Surgeon of that place.

THE variolous epidemic which so generally, and in many instances so fatally, prevailed here in 1825, and against which vaccination made such a bold and successful stand, broke out the latter end of July or beginning of August. It was of the confluent kind, the symptoms running very high; it had no particular or unusual character: it was a severe confluent small-pox, the phenomena of which I need not describe.

The entire population of Bury is something more than ten thousand. In November, 1824, the Court of Guardians ordered a general inoculation for the cow-pox, and the number of poor allotted to me for vaccination, (having one of the five medical districts,) was 149; added to which were several out-settlers, amounting to twelve. Of this number, from strong prejudice, little more than half submitted to be vaccinated. Ten cases of small-pox after vaccination fell to my share: the eruptive fever was generally severe, in three or four cases as much so as in confluent small-pox; the pustules few, excepting in one case. Indeed, the disease appeared to have spent itself in supporting the eruptive fever, and to have had no power left to produce or perfect the pustules, which in every instance, excepting this one, did not stand over the fifth day. The pustules had not the character either of small-pox or cow-pox, they more resembled the chicken-pock; there was no secondary fever; and but slight sore-throat, in three or four instances only.

The severe case I have alluded to occurred in a young man of scrofulous habit, about nineteen years of age, who had been vaccinated twelve years before by a respectable surgeon. The eruptive fever was very intense; the pustules extremely confluent, particularly upon the face, flat and small, accompanied on the second day by a purple eruption, amounting almost to the purpura hemorrhagica. On the third day, bleeding gums supervened, with fetid breath, severe sore-throat, and great anxiety. These symptoms continued through the fourth and fifth days. On the sixth, they had somewhat abated. On the evening of the seventh day of the eruption, the pock might be said to be about to turn. *Turning* it could not properly be called, where there had been nothing to be termed suppuration. On the eighth day, the patient was convalescent.

This man surely had been saved, the period of his disease shortened, and the formation of a vast quantity of pus prevented, by his previous vaccination. From an examination of his arm, upon which there is a deep, large, cup-shaped scar, it appears he must have had, when vaccinated, much common inflammation, with suppuration of the cellular texture, under the vaccine pustule; a circumstance I always consider as destructive of the perfect security which cow-pox affords; and, in most instances of failure of perfect protection, I have generally found such a scar.

I will briefly add, that, where I have been induced, by the fears of my patients, to revaccinate as a test of their perfect security, I have invariably found a very extensive local inflammation of a mixed character, with great soreness and enlargement of the axillary glands, high constitutional irritation, and in several instances vesications threatening sphacelus, with an irregularly formed pustule, pouring out a semi-ichorous and ill-concocted acrid discharge.

*Bury; Feb.* 7, 1826.

